# Objective Sleep Assessment in Systemic Mastocytosis: Polysomnographic Findings from a Single-Center Observational Study

**DOI:** 10.3390/jcm15156068

**Published:** 2026-08-04

**Authors:** Hatice Serpil Akten, Şeyma Aykaç, Burhanettin Uludağ, Ceyda Tunakan Dalgıç, Reyhan Gümüşburun, Sinem İnan, Kasım Okan, Züleyha Galata, Eda Aslan, Ecem Ay, Asuman Camyar, Hasibe Aytaç, Meryem Demir, Onurcan Yıldırım, Gökten Bulut, Umitcan Ates, Türkan Dizdar Canbaz, Meryem İrem Toksoy Sentürk, Seda Karaaslan Yetemen, Aytül Zerrin Sin, E. Nihal Mete Gokmen

**Affiliations:** 1 Division of Allergy, and Clinical Immunology, Department of Internal Medicine, Faculty of Medicine, Ege University, Izmir 35100, Türkiye; aktenhaticeserpil@gmail.com (H.S.A.); dr_ceydat@yahoo.com (C.T.D.); reyhangumusburun@gmail.com (R.G.); zuleyhagalata61@gmail.com (Z.G.); edaarslan_91@hotmail.com (E.A.); ecem.ay@live.com (E.A.); umitcanates@gmail.com (U.A.); turkan88dizdar@gmail.com (T.D.C.); irem.toksoy@yahoo.com (M.İ.T.S.); seda.karaaslan95@gmail.com (S.K.Y.); aytulsin@yahoo.com (A.Z.S.); 2 Department of Allergy and Clinical Immunology, Dr. Suat Seren Chest Diseases and Surgery Training and Research Hospital, Health Sciences University, Izmir 35220, Türkiye; hasibeuchkun@hotmail.com; 3 Department of Neurology, Faculty of Medicine, Ege University, Izmir 35100, Türkiye; symaykac@gmail.com (Ş.A.); burhanettin.uludag@gmail.com (B.U.); 4 Department of Allergy and Immunology, Izmir City Hospital, Izmir 35540, Türkiye; drsineminan@gmail.com; 5 Department of Allergy and Immunology, Ordu University Training and Research Hospital, Ordu 52200, Türkiye; kasimokan55@gmail.com; 6 Department of Immunology and Allergy Diseases, Izmir Bakircay University Cigli Regional Training and Research Hospital, Izmir 35660, Türkiye; asuerden@yahoo.com; 7 Department of Allergy and Immunology, Kartal Dr. Lütfi Kırdar City Hospital, Istanbul 34865, Türkiye; meryem_durgay@hotmail.com; 8 Department of Allergy and Immunology, Osmaniye State Hospital, Osmaniye 80000, Türkiye; onurcanyildirim@hotmail.com; 9 Department of Allergy and Immunology, Balıkesir Atatürk City Hospital, Balıkesir 10100, Türkiye; goktenbulut58@gmail.com

**Keywords:** systemic mastocytosis, polysomnography, obstructive sleep apnea, sleep architecture, brain fog, mast cells

## Abstract

**Background/Objectives**: Systemic mastocytosis (SM) is a rare clonal mast cell disorder associated with mediator-related symptoms and a broad range of neurocognitive and sleep-related complaints. Fatigue, insomnia, and brain fog are frequently reported in clinical practice, but objective polysomnographic data in this patient population remain limited. As overweight and obesity are established contributors to sleep-disordered breathing, polysomnography (PSG) findings in patients with SM should be interpreted with caution when a control group is not available. This study aimed to characterize sleep architecture, sleep continuity, and sleep-disordered breathing in adult patients with SM who underwent PSG. **Methods**: This single-center observational study included nine adult patients diagnosed with SM from the Ege University Mastocytosis Database between January 2023 and January 2024. No control group was included. All patients underwent overnight laboratory PSG after discontinuation of antihistamine therapy for at least seven days. Demographic characteristics, SM subtype, baseline serum tryptase levels, Epworth Sleepiness Scale (ESS) scores, and brain fog-like cognitive complaints were recorded. PSG parameters included total sleep time, sleep efficiency, sleep onset latency, wake after sleep onset, rapid eye movement (REM) latency, sleep stage distribution, arousal burden, apnea–hypopnea index (AHI), oxygenation parameters, and periodic limb movements. **Results**: The study included four women and five men, with a median age of 50 years. Eight patients had a body mass index (BMI) ≥ 25 kg/m^2^, indicating that the cohort was predominantly overweight or obese. The most common SM subtype was indolent SM. The median baseline serum tryptase level was 55.9 ng/mL, and the median ESS score was 9. Only two patients had excessive daytime sleepiness according to ESS > 10, whereas six patients reported brain fog-like complaints. Median sleep efficiency was 91.4%, total sleep time was 344 min, sleep onset latency was 9 min, and wake after sleep onset was 11 min. Median REM latency was 128.5 min. Sleep stage distribution showed median N1, N2, N3, and REM sleep proportions of 3%, 54%, 26%, and 16.7%, respectively. One patient had no observed REM sleep, and two patients had no measurable N3 sleep. The median number of arousals was 55. The median AHI was 8.2 events/hour. Although eight of nine patients met PSG criteria for obstructive sleep apnea (OSA), including five with mild and three with severe disease, this finding should be interpreted in the context of the high prevalence of overweight/obesity in the cohort. All patients with BMI ≥ 25 kg/m^2^ had OSA, whereas the only normal-weight patient did not. **Conclusions**: In this single- center observational study, PSG demonstrated frequent sleep-disordered breathing, REM-related changes, and variable arousal burden in patients with SM; however, these findings cannot be separated from the predominance of overweight/obesity and the absence of a control group. Brain fog-like complaints were common, although excessive daytime sleepiness was present in only a minority of patients. Because most patients were overweight or obese and no control group was included, these findings cannot be attributed directly to SM. However, the results suggest that objective sleep assessment may be useful in selected patients with SM, particularly when fatigue, non-restorative sleep, or cognitive complaints are present. Larger controlled studies with BMI-matched groups are needed to clarify the relationship between SM, mast cell mediator activity, and sleep abnormalities.

## 1. Introduction

Systemic mastocytosis (SM) is a rare clonal mast cell disorder characterized by the accumulation of abnormal mast cells in extracutaneous tissues and organs. According to the current classification, SM includes non-advanced forms, such as bone marrow mastocytosis (BMM), indolent systemic mastocytosis (ISM), and smoldering systemic mastocytosis (SSM), as well as advanced forms, including aggressive systemic mastocytosis (ASM), SM with an associated hematologic neoplasm (SM-AHN), and mast cell leukemia (MCL). Diagnosis is based on clinical, morphologic, immunophenotypic, and molecular findings that reflect mast cell burden, organ involvement, and the need for disease-modifying or cytoreductive therapy [1,2,3].

Mast cell activation in SM may occur spontaneously or be triggered by external factors such as medications or insect venom. Patients often experience a broad spectrum of mediator-related symptoms, including pruritus, flushing, headaches, presyncope or syncope, gastrointestinal complaints, and anaphylaxis. In addition to these systemic manifestations, cognitive and neuropsychiatric symptoms, including brain fog, memory difficulties, impaired concentration, anxiety, depression, insomnia, sleep paralysis, and vivid nightmares, have also been reported and may substantially impair quality of life [4,5,6]. Circadian rhythm and sleep–wake disturbances have been linked to mood disorders, providing a broader mechanistic context for the overlap between affective symptoms and sleep-related complaints [6]. However, brain fog in clinical cohorts is often documented as a patient-reported or clinically recorded complaint rather than as an objectively measured neurocognitive outcome, and this distinction is important when interpreting cognitive symptoms in SM. These observations suggest that the clinical burden of SM may extend beyond classical allergic and anaphylactic manifestations.

Several biological pathways may link mast cell activity with sleep–wake regulation. Brain-resident mast cells have been described in regions involved in arousal and behavioral state control, and their mediators may interact with neuronal and inflammatory pathways relevant to sleep regulation [7]. Among these mediators, histamine is a major wake-promoting neurotransmitter; histaminergic activity is highest during wakefulness, decreases during non-rapid eye movement (NREM) sleep, and is minimal during rapid eye movement (REM) sleep [8,9,10,11]. Cytokines and other inflammatory mediators may also modulate sleep continuity and sleep architecture [12]. Experimental studies further suggest that mast cell activity may influence behavioral states and stress-related sleep disturbance [8,13]. However, these data remain largely experimental, and their relevance to polysomnography (PSG) findings in patients with SM should be interpreted as hypothesis-generating rather than causal.

Sleep architecture is conventionally organized into NREM and REM sleep. NREM sleep includes stages N1, N2, and N3, with N3 representing slow-wave sleep, whereas REM sleep is involved in dreaming, memory consolidation, and emotional processing [14]. Accordingly, objective assessment of sleep architecture may be relevant in patients with SM who report fatigue, non-restorative sleep, or cognitive complaints; however, such findings must be interpreted in the context of established determinants of sleep quality and recognized risk factors for sleep-disordered breathing, including age, body mass index, and comorbid conditions [14,15,16,17].

Despite the frequent reporting of fatigue, brain fog, insomnia, and other neurocognitive symptoms in SM, objective sleep assessment in this patient population remains limited. PSG provides a comprehensive evaluation of sleep architecture, sleep efficiency, respiratory events, oxygenation, and arousal burden. However, data on polysomnographic findings in patients with SM are scarce.

Therefore, this single-center observational study aimed to characterize polysomnographic findings in adult patients with SM and to describe sleep-disordered breathing, sleep architecture, and sleep continuity in this rare patient population. Given its descriptive design and the absence of a control group, the study was intended to provide hypothesis-generating objective sleep data rather than to determine whether PSG abnormalities are specifically attributable to SM. The findings may support a more structured evaluation of sleep-related complaints in SM and inform the design of future controlled studies.

## 2. Methods

### 2.1. Participants

During the study period, 16 patients with SM were screened for eligibility. Of these, 7 patients were not included: two patients had declined the recommended PSG during routine clinical evaluation, three patients were unable to discontinue antihistamine therapy before PSG, and two patients underwent an attempted PSG recording but could not complete the examination because they were unable to sleep during the recording night. Finally, nine patients with complete overnight PSG recordings and available clinical data were included in the analysis.

Patients were identified from the Ege University Mastocytosis Database and screened on the basis of clinical and PSG records obtained between January 2023 and January 2024. Eligible patients were required to meet the World Health Organization 5th edition diagnostic criteria for mastocytosis [1], be between 18 and 80 years of age, and have undergone overnight in-laboratory PSG as part of their clinical sleep evaluation. Patients who were unable to discontinue antihistamine therapy for at least seven days before PSG were not eligible to undergo PSG under the clinical preparation protocol and were therefore not included. Because the study was based on existing clinical records and PSG reports, only patients with complete PSG data and available clinical information were included in the final analysis.

Demographic and clinical data, including age, sex, body mass index (BMI), and SM subtype, were recorded. Baseline serum tryptase levels were obtained for all patients. Daytime sleepiness was assessed using the Epworth Sleepiness Scale (ESS), with scores > 10 considered indicative of excessive daytime sleepiness. Patients were also evaluated for symptoms commonly described as “brain fog”, defined as a cluster of cognitive complaints involving reduced memory, attention, and mental clarity [15]. Brain fog was recorded as a clinically reported symptom based on patient history and clinical documentation rather than being assessed with a validated neurocognitive instrument. Therefore, it was analyzed as a subjective, clinically recorded complaint rather than as an objective measure of cognitive impairment. Concomitant medications recorded at the time of the clinical evaluation preceding PSG were also extracted from the clinical records and summarized in Supplementary Table S1.

### 2.2. Study Design and Procedure

This was a single-center observational study involving patients with SM who underwent PSG at the Ege University Department of Neurology Sleep Laboratory. The study comprised a retrospective analysis of existing clinical records and PSG reports obtained between January 2023 and January 2024. All PSG recordings were performed after discontinuation of antihistamine therapy for at least seven days and were conducted in accordance with the standards of the American Academy of Sleep Medicine (AASM) [18]. The seven-day antihistamine washout was applied as part of the clinical PSG preparation procedure only in patients for whom treatment discontinuation was considered clinically safe and tolerable.

The study was approved by the local ethics committee and was conducted in accordance with the principles of the Declaration of Helsinki. The ethics approval covered the retrospective use of anonymized clinical records and PSG data collected before the approval date. Patient data were analyzed anonymously using existing clinical records and PSG reports.

### 2.3. Polysomnography Protocol

Each overnight PSG study included electroencephalography (EEG), electrooculography (EOG), electromyography (EMG), electrocardiography (ECG), nasal airflow measurement, thoracic and abdominal respiratory effort monitoring, pulse oximetry for oxygen saturation, and body position recording. Sleep stages and respiratory events were manually scored by a certified sleep technologist according to AASM criteria, version 2.4 [18]. All PSG recordings were scored by a single certified sleep technologist, and no independent second scoring or inter-rater verification was performed.

The following polysomnographic parameters were evaluated: total sleep time (TST), sleep efficiency (SE), sleep onset latency (SOL), REM latency, wake after sleep onset (WASO), sleep stage distribution including N1, N2, N3, and REM sleep, total number of arousals, apnea–hypopnea index (AHI), oxygen desaturation parameters, and periodic limb movements during sleep, when present.

Obstructive sleep apnea (OSA) severity was classified according to AHI as no OSA (<5 events/hour), mild OSA (5–14.9 events/hour), moderate OSA (15–29.9 events/hour), or severe OSA (≥30 events/hour).

### 2.4. Statistical Analysis

Descriptive statistics were used to summarize demographic and polysomnographic data. Given the small sample size and exploratory nature of the study, no formal hypothesis testing was performed. Continuous variables were presented as median (min–max), and categorical variables as n (%). As an exploratory analysis, Spearman correlation coefficients were calculated between BMI, serum tryptase levels, ESS scores, and selected PSG parameters, including AHI, sleep efficiency, N3 sleep percentage, REM sleep percentage, sleep latency, and REM latency. Given the very small sample size, these analyses were considered descriptive and hypothesis-generating only, and no inferential conclusions were drawn.

### 2.5. Ethical Approval

This study received ethical approval from the Ege University Faculty of Medicine Ethics Committee for Medical Research (Approval date: 7 March 2024; Reference number: 24-3T/68).

## 3. Results

Nine patients diagnosed with SM were included in this observational study. The cohort consisted of four women and five men, with a median age of 50 years (range, 34–73 years). Eight patients had a BMI ≥ 25 kg/m^2^, including three overweight patients, four patients with obesity, and one patient with morbid obesity. ISM was recorded in five patients. Other subtypes included BMM in two patients, ASM in one patient, and SM-AHN in one patient. The median baseline serum tryptase level before PSG was 55.9 ng/mL (range, 17.6–127 ng/mL). Baseline demographic and laboratory characteristics are summarized in Table 1.

The median ESS score before PSG was 9 (range, 0–11). Only two patients had scores > 10, indicating excessive daytime sleepiness. Cognitive complaints compatible with brain fog, including difficulties with memory, attention, or concentration, were reported by six patients.

Polysomnographic findings are presented in Table 2. Median SE was 91.4% (range, 73.5–96.9%), and median TST was 344 min (range, 266–475 min). Median SOL was 9 min (range, 1–42 min), and median WASO was 11 min (range, 4–28.5 min). REM sleep was not observed in one patient; therefore, REM latency was calculated for the remaining eight patients. The median REM latency was 128.5 min (range, 3–279 min). One patient had a REM latency of 3 min, consistent with a sleep-onset REM period. This finding was noted as an individual observation, but no diagnostic inference was made because of the descriptive design and the absence of complementary clinical or neurophysiological assessment for central disorders of hypersomnolence.

**Figure 1:**
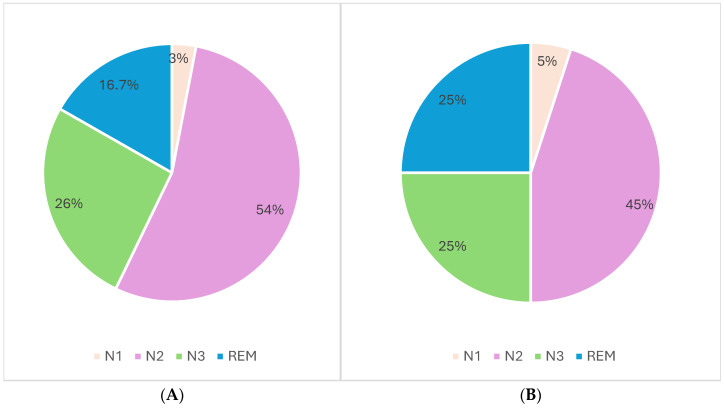
Sleep-stage distribution in the study cohort and published adult reference values. (**A**) Median sleep-stage proportions in the study cohort. Percentages represent median values and may not sum to 100% due to rounding. (**B**) Published adult reference values derived from Ohayon et al. [16], shown for descriptive context.

Evaluation of sleep architecture showed that N2 sleep accounted for the largest proportion of total sleep time, with a median value of 54% (range, 46–80%). Median N1 sleep was 3% (range, 1.3–17%), median N3 sleep was 26% (range, 0–40%), and median REM sleep was 16.7% (range, 0–26%). Two patients had no measurable N3 sleep, and one patient had no observed REM sleep. Sleep-stage distribution in the study cohort is displayed together with published adult reference values in Figure 1 to provide descriptive context; no formal comparison with these reference values was performed.

**Figure 2:**
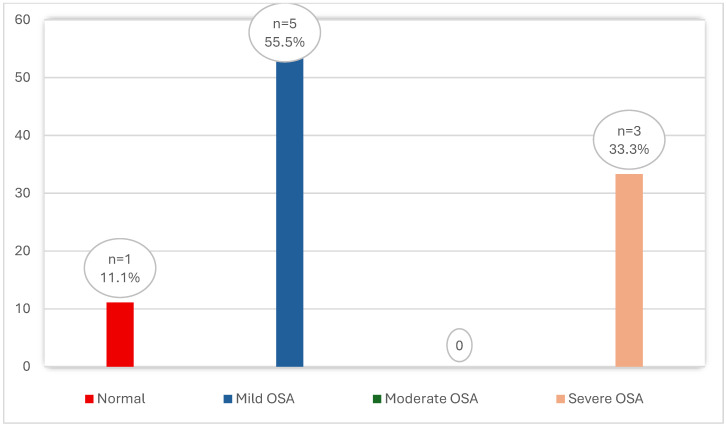
Distribution of obstructive sleep apnea severity according to apnea–hypopnea index.

**Figure 3:**
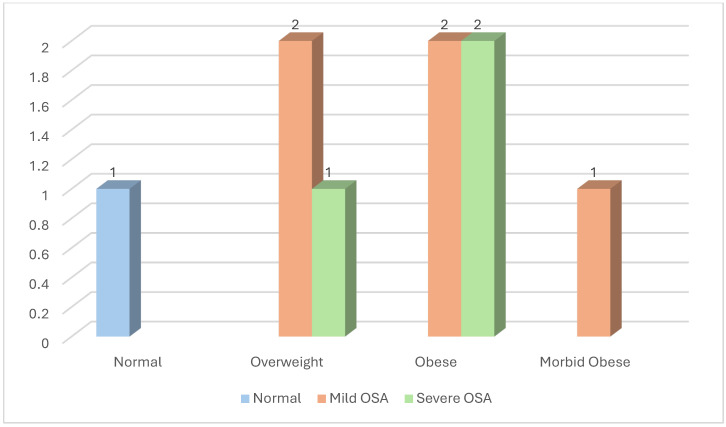
Distribution of obstructive sleep apnea severity according to BMI category.

Sleep-disordered breathing was observed in most patients in this cohort. The median AHI was 8.2 events/hour (range, 2.2–100.3 events/hour). Based on the AHI, eight of nine patients met the criteria for OSA. Five patients had mild OSA, three patients had severe OSA, and one patient had an AHI below the diagnostic threshold for OSA. No patient had moderate OSA. The highest AHI value was 100.3 events/hour, indicating a substantial respiratory-event burden in one patient. This patient also had a sleep efficiency of 96.9%; thus, in this individual recording, a high respiratory-event burden coexisted with preserved sleep efficiency. All patients with BMI ≥ 25 kg/m^2^ had OSA. Given the composition and size of the cohort, this observation was considered descriptive and was not interpreted as evidence of an association between BMI and OSA. The distribution of OSA severity is shown in Figure 2, and the distribution of OSA severity according to BMI category is shown in Figure 3.

The median total number of arousals was 55 (range, 0–125), and the median number of periodic limb movements was 0 (range, 0–34). Given the very small sample size and descriptive design, no inferential statistical assessment of associations between SM subtype, serum tryptase level, daytime sleepiness, and OSA severity was performed. Therefore, the present dataset does not allow any conclusion regarding these potential relationships. Individual demographic and polysomnographic findings are presented in Table 3. Exploratory Spearman correlation analyses between BMI, serum tryptase levels, ESS scores, and selected PSG parameters are presented in Supplementary Table S2. Given the very small sample size, these analyses were interpreted descriptively only and were not used to draw inferential conclusions.

## 4. Discussion

This single-center observational study provides an objective polysomnographic characterization of sleep architecture, sleep continuity, and sleep-disordered breathing in patients with SM. The PSG findings described in this cohort included OSA, REM-related observations, variable arousal burden, and brain fog-like symptoms. Because the study was descriptive, uncontrolled, and based on a very small sample, these observations should not be interpreted as evidence of disease-specific PSG abnormalities in SM. Notably, all patients with BMI ≥ 25 kg/m^2^ had OSA, whereas the only normal-weight patient did not. Therefore, the respiratory findings should be interpreted primarily in the context of established OSA risk factors, particularly overweight and obesity, rather than as evidence of an SM-specific association. These findings are relevant because fatigue, sleep complaints, and cognitive symptoms are commonly reported in SM but have rarely been characterized using PSG.

SM is a rare clonal mast cell disorder characterized by the accumulation of abnormal mast cells in extracutaneous tissues [1,2,3,4]. The clinical burden of the disease is not limited to anaphylaxis, flushing, pruritus, or gastrointestinal symptoms. Many patients also report non-specific but disabling symptoms such as fatigue, impaired concentration, memory difficulties, mood changes, and sleep-related complaints [5,19,20,21]. These symptoms may be multifactorial and are not always reflected by standard laboratory or disease-burden markers. PSG may therefore provide complementary information by objectively assessing sleep architecture, respiratory events, and sleep fragmentation. However, in the present cohort, the high frequency of OSA cannot be separated from the predominance of overweight and obesity.

Several biological mechanisms may link mast cell activation and sleep regulation. Mast cells release several mediators, including histamine, prostaglandins, cytokines, and chemokines, which may influence neuronal activity, arousal, and inflammatory pathways [7,12]. Histamine is one of the key wake-promoting neurotransmitters and plays an important role in the regulation of the sleep–wake cycle. During wakefulness, histaminergic activity promotes cortical arousal, whereas histaminergic neuronal activity decreases during NREM sleep and is minimal during REM sleep [8,9,10,11]. Experimental studies have also suggested that brain-resident mast cells may contribute to behavioral state regulation and sleep quality [7,8]. In addition, Chikahisa et al. reported that mast cell involvement may be associated with sleep disturbance in a chronic mild stress model, and that inhibition of mast cell function improved sleep-related alterations [13]. These findings provide biological context but do not establish that mast cell activity caused the PSG observations in our cohort.

REM-related findings were noted in several patients in this cohort. REM sleep was not observed in one patient, and REM latency was prolonged in some others. REM sleep normally accounts for a limited but important proportion of total sleep time and has been associated with dreaming, emotional processing, and memory-related functions [16,17,22]. However, REM-related findings in this cohort must be interpreted in the context of comorbid sleep-disordered breathing. OSA and sleep fragmentation may independently alter sleep architecture, including REM sleep and slow-wave sleep. Accordingly, the REM-related observations should not be considered an SM-specific PSG pattern. In our cohort, six of nine patients reported difficulties with memory, attention, or mental clarity, whereas only two had ESS-defined excessive daytime sleepiness. This suggests that brain fog-like complaints and daytime sleepiness did not fully overlap. However, brain fog was clinically recorded rather than assessed using a validated neurocognitive instrument; therefore, no conclusion can be drawn regarding objective cognitive impairment.

Although median sleep efficiency was relatively preserved, the number of arousals varied across patients. Recurrent arousals may contribute to non-restorative sleep, fatigue, and impaired daytime functioning, even when total sleep time and sleep efficiency appear acceptable. In the present study, arousal burden should be interpreted cautiously because arousals may reflect respiratory events, comorbid sleep-disordered breathing, medication effects, antihistamine withdrawal, or other non-SM-related factors. Mast cell mediators, particularly histamine and inflammatory cytokines, may theoretically promote micro-arousals or lighter sleep [8,9,10,11,12], but this mechanism cannot be confirmed from the present data. These findings therefore remain descriptive and hypothesis-generating.

OSA was identified in eight of nine patients. Five patients had mild OSA and three had severe OSA. This observation should be interpreted primarily in the context of the cohort’s BMI distribution, because eight patients were overweight or obese and no BMI-matched control group was included. Therefore, the present data cannot establish that SM itself increases the risk of OSA. Nevertheless, the observation is clinically relevant because sleep-disordered breathing is treatable and may contribute to fatigue, cognitive complaints, and impaired quality of life. In selected patients with SM who report fatigue, non-restorative sleep, snoring, witnessed apneas, or cognitive complaints, screening for sleep-disordered breathing may therefore be appropriate.

The possible contribution of mast cell-related mechanisms to sleep-disordered breathing remains uncertain. In addition to obesity and anatomical factors, mediator-related mucosal edema, inflammation, increased secretions, or tissue remodeling could theoretically affect upper airway patency. Similar mechanisms have been suggested in mast cell activation-related conditions, but evidence remains limited [23]. In the present cohort, however, all patients with BMI ≥ 25 kg/m^2^ had OSA, which suggests that conventional risk factors likely played an important role. Therefore, the observed sleep-disordered breathing should not be interpreted as evidence of an independent association between SM and OSA. Overall, the study supports the potential clinical value of objective sleep evaluation in selected patients with SM but does not demonstrate that SM independently contributes to OSA or other PSG abnormalities.

This study has several limitations. The sample size was small, reflecting both the rarity of SM and the practical difficulty of performing PSG after antihistamine discontinuation. The absence of a control group, particularly a BMI-matched control group, limits the ability to determine whether the observed PSG findings differ from those expected in comparable individuals without SM. The descriptive design also precludes causal interpretation and robust subgroup comparisons. Brain fog was assessed clinically rather than using a validated neurocognitive instrument, and subjective sleep burden was assessed only with the ESS. Validated insomnia and sleep-quality instruments, such as the Insomnia Severity Index or Pittsburgh Sleep Quality Index, were not used [24]. PSG scoring was performed by a single certified technologist without inter-rater verification. The seven-day antihistamine washout may also have influenced sleep continuity and sleep architecture by removing sedative effects or increasing mediator-related symptoms. Concomitant medications, including antidepressants, biologic therapy, hydroxyurea, and mast-cell-directed treatments, may also have affected PSG findings. Finally, selection bias is possible because only patients who were able to discontinue antihistamines and complete PSG were included. Therefore, the cohort may not represent the broader SM population.

## 5. Conclusions

In this single-center observational study of patients with SM, PSG showed frequent sleep-disordered breathing, REM-related observations, and variable arousal burden; however, these findings cannot be separated from the high rate of overweight and obesity in the cohort and the absence of a BMI-matched control group. Brain fog-like complaints were common, although most patients did not have excessive daytime sleepiness according to the ESS. Therefore, the present findings should be interpreted as descriptive and hypothesis-generating rather than as evidence of SM-specific sleep abnormalities. Despite these limitations, the results support considering sleep disorders during the clinical evaluation of selected patients with SM, particularly those with fatigue, non-restorative sleep, snoring, witnessed apneas, or cognitive complaints. Further studies with larger samples, validated cognitive and sleep-quality assessments, and BMI-matched control groups are needed to better define these associations.

## Figures and Tables

**Table 1:** Demographic and laboratory characteristics of patients with systemic mastocytosis.

Variables	
Age; years, median (min–max)	50 (34–73)
Sex; n (%)	
Female	4 (44.4)
Male	5 (55.6)
BMI; n (%)	
Normal	1 (11.1)
Overweight	3 (33.3)
Obese	4 (44.4)
Morbid obesity	1 (11.1)
SM Type; n (%)	
ISM	5 (55.6)
BMM	2 (22.2)
ASM	1 (11.1)
SM-AHN	1 (11.1)
Patients experiencing brain fog; n (%)	6 (66.7)
Baseline serum tryptase before PSG, ng/mL; median (min–max)	55.90 (17.6–127)
Epworth Sleepiness Scale; median (min–max)	9 (0–11)

SM: Systemic Mastocytosis, BMI: Body Mass Index, PSG: Polysomnography, BMM: Bone Marrow Mastocytosis, ISM: Indolent SM, ASM: Aggressive SM, SM-AHN: SM-Associated Hematologic Neoplasm.

**Table 2:** Polysomnographic findings of the study cohort.

Variables	Median (Min–Max)
SE, %	91.4 (73.5–96.9)
TST, minutes	344 (266–475)
SOL, minutes	9 (1–42)
REM latency, minutes	128.5 (3–279)
WASO, minutes	11 (4–28.5)
N1 sleep, %	3 (1.3–17)
N2 sleep, %	54 (46–80)
N3 sleep, %	26 (0–40)
REM sleep, %	16.7 (0–26)
Number of arousals, count	55 (0–125)
AHI, events/hour	8.2 (2.2–100.3)
Number of PLM, count	0 (0–34)

TST: Total Sleep Time, SE: Sleep Efficiency, SOL: Sleep Onset Latency, REM: Rapid Eye Movement, WASO: Wake After Sleep Onset, AHI: Apnea–Hypopnea Index, PLM: Periodic Limb Movement.

**Table 3:** Individual demographic and polysomnographic characteristics of patients.

Patient No.	Sex	Age	BMI (kg/m^2^)	SM Type	Serum Tryptase (ng/mL)	ESS	SE (%)	SOL (minutes)	REM Latency (minutes)	WASO (minutes)	N1 (%)	N2 (%)	N3 (%)	REM (%)	AHI (Events/hour)
1	M	34	31	BMM	126	8	96.9	6	73.5	4	1.3	52.4	29.4	16.7	100.3
2	F	43	29.4	ISM	127	11	78.8	3	194	7	3	55	31	11	6.1
3	M	43	27.7	ISM	96.9	2	73.5	42	134	9	2	46	26	26	9
4	F	63	30.9	ISM	58.9	10	91.4	1	NA	11	2	68	30	0	47.2
5	M	50	19.2	ISM	24.2	9	94.7	9	91	10	4	54	25	17	2.2
6	M	73	28.3	ASM	55.9	3	92.3	12	123	13	6	74	2	18	46.1
7	F	51	34.1	ISM	37.5	9	92.5	9	142	11	3	49	40	8	8.2
8	F	68	40	SM-AHN	23.3	9	88.5	16.3	3	28.5	3	51.2	25.4	20.3	6.4
9	M	35	31.3	BMM	17.6	0	87.4	13	279	23	17	80	0	3	5.9

SM: Systemic Mastocytosis, BMI: Body Mass Index, BMM: Bone Marrow Mastocytosis, ISM: Indolent SM, ASM: Aggressive SM, SM-AHN: SM-Associated Hematologic Neoplasm, ESS: Epworth Sleepiness Scale, SE: Sleep Efficiency, SOL: Sleep Onset Latency, REM: Rapid Eye Movement, WASO: Wake After Sleep Onset, AHI: Apnea–Hypopnea Index. NA: not applicable because REM sleep was not observed during the PSG recording.

## Data Availability

Data and materials are available upon request from the corresponding authors.

## Supplementary Materials

The following supporting information can be downloaded at: https://www.mdpi.com/article/10.3390/jcm15156068/s1, Table S1. Concomitant medications recorded before PSG evaluation; Table S2. Exploratory Spearman correlations between clinical variables and selected PSG parameters.
