# Supplementary material for: Objective Sleep Assessment in Systemic Mastocytosis: Polysomnographic Findings from a Single-Center Observational Study
Source: J Clin Med. 2026 Aug 4;15(15):6068. doi: 10.3390/jcm15156068 (PMC13467162; doi:10.3390/jcm15156068)
Supplement: Supplementary file 1 [file jcm-15-06068-s001.zip › jcm-4407874-supplementary.pdf]

Supplementary Table S1. Concomitant medications recorded before PSG evaluation.

Patient No.	Concomitant medications
1	Famotidine, ketotifen
2	None
3	None
4	Montelukast, famotidine
5	Famotidine, escitalopram
6	None
7	Denosumab, vitamin D, calcium
8	None
9	Hydroxyurea, desloratadine, montelukast, famotidine, duloxetine, denosumab

Supplementary Table S2. Exploratory Spearman correlations between clinical variables and selected PSG parameters.

Clinical variable	PSG parameter	n	Spearman's rho	p value
BMI	AHI	9	0.083	0.831
BMI	Sleep efficiency	9	0.050	0.898
BMI	N3 sleep %	9	0.200	0.606
BMI	REM latency	8	−0.024	0.955
BMI	Sleep latency	9	0.059	0.881
BMI	REM sleep %	9	-0.317	0.406
Serum tryptase	AHI	9	0.533	0.139
Serum tryptase	Sleep efficiency	9	-0.083	0.831
Serum tryptase	N3 sleep %	9	0.600	0.088
Serum tryptase	REM latency	8	0.024	0.955
Serum tryptase	Sleep latency	9	-0.494	0.177
Serum tryptase	REM sleep %	9	0.050	0.898
ESS	AHI	9	-0.051	0.897
ESS	Sleep efficiency	9	0.102	0.795
ESS	N3 sleep %	9	0.678	0.045
ESS	REM latency	8	−0.195	0.643
ESS	Sleep latency	9	-0.715	0.030
ESS	REM sleep %	9	-0.305	0.425

Note: These analyses were exploratory and descriptive only. REM latency correlations included eight patients because REM sleep was not observed in one patient. Given the very small sample size and the multiple exploratory analyses performed, p values should not be interpreted as confirmatory evidence of association. **Abbreviations:** BMI, body mass index; AHI, apnea–hypopnea index; ESS, Epworth Sleepiness Scale; PSG, polysomnography; REM, rapid eye movement.
